# Targeting extracellular glycans: tuning multimeric boronic acids for pathogen-selective killing of *Mycobacterium tuberculosis*†
†Electronic supplementary information (ESI) available. See DOI: 10.1039/c9sc00415g


**DOI:** 10.1039/c9sc00415g

**Published:** 2019-05-16

**Authors:** Collette S. Guy, Matthew I. Gibson, Elizabeth Fullam

**Affiliations:** a School of Life Sciences , University of Warwick , CV4 7AL , UK . Email: e.fullam@warwick.ac.uk; b Department of Chemistry , University of Warwick , Coventry , CV4 7AL , UK . Email: m.i.gibson@warwick.ac.uk; c Warwick Medical School , University of Warwick , Coventry , CV4 7AL , UK

## Abstract

Innovative chemotherapeutic agents that are active against *Mycobacterium tuberculosis* (*Mtb*) are urgently required to control the tuberculosis (TB) epidemic. The *Mtb* cell envelope has distinct (lipo)polysaccharides and glycolipids that play a critical role in *Mtb* survival and pathogenesis and disruption of pathways involved in the assembly of the *Mtb* cell envelope are the primary target of anti-tubercular agents. Here we introduce a previously unexplored approach whereby chemical agents directly target the extracellular glycans within the unique *Mtb* cell envelope, rather than the intracellular biosynthetic machinery. We designed and synthesised multimeric boronic acids that are selectively lethal to *Mtb* and function by targeting these structurally unique and essential *Mtb* cell envelope glycans. By tuning the number of, and distance between, boronic acid units high selectivity to *Mtb*, low cytotoxicity against mammalian cells and no observable resistance was achieved. This non-conventional approach may prevent the development of drug-resistance and will act as a platform for the design of improved, pathogen-specific, next generation antibiotics.

## Abstract 2

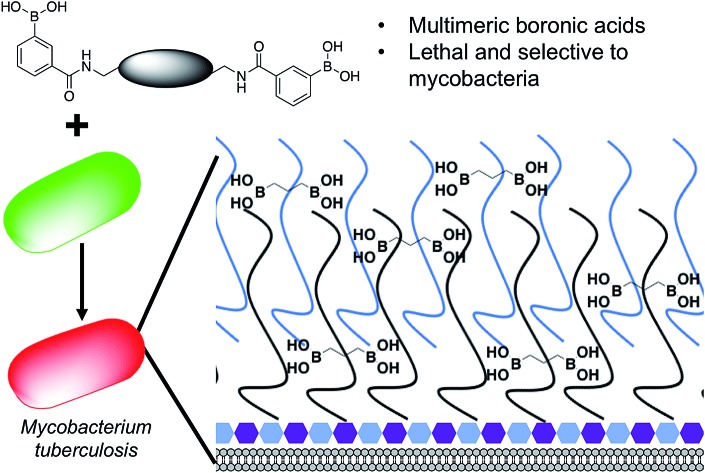
Innovative chemotherapeutic agents that are active against *Mycobacterium tuberculosis* (*Mtb*) are urgently required to control the tuberculosis (TB) epidemic.

## Introduction

*Mycobacterium tuberculosis*, the causative agent of tuberculosis (TB), is the world's leading cause of death from a single infectious agent claiming the lives of 1.7 million people annually.^1^ The incidences of drug resistant strains of *Mtb* are increasing at an alarming rate and include the emergence of *Mtb* strains that are not treatable with any of the current antibiotic regimens.^1^ Consequently, there is an urgent need for the development of innovative, next-generation anti-tubercular treatments that function by distinct mechanisms compared to the current drugs available. *Mtb* possesses a distinctive cell envelope that is uniquely complex and rich in a diverse range of unusual carbohydrates and lipids.^2^ The cell envelope has a fundamental role in the pathogenesis and virulence of *Mtb* and provides a highly efficient permeability barrier that prevents intracellular access to many antibiotics and severely complicates anti-tubercular treatment regimens. The core of the *Mtb* cell wall is comprised of three main components: a cross-linked peptidoglycan (PG) network, a highly branched arabinogalactan (AG) with both arabinose and galactose found in the furanose form, and long chain (C_60–90_) mycolic acids.^3^ The outer ‘myco-membrane’ contains a large array of distinct glycolipids and lipoglycans that are interspersed within this core and include phosphatidylinositol mannosides (PIMS), phthiocerol dimycocerosates (PDIMs), lipomannan (LM), lipoarabinomannan (LAM), mannose-capped LAM (ManLAM), sulfolipids and trehalose mono- and di-mycolates (TMM, TDM).^4^ The final component of the *Mtb* envelope is an outer capsule composed of polysaccharides, predominantly α-glucan, and proteins.^5^,^6^ Intriguingly, many of these carbohydrates are specific to the *Mycobacterium* genus and are found in unusual conformations with distinct glycosidic linkages. Molecular pathways directly involved in the biosynthesis of the *Mtb* cell envelope have proven to be especially vulnerable to chemotherapeutic agents and include the front-line drugs isoniazid^7^ and ethambutol^8^ and second-line drugs ethionamide^7^ and d-cycloserine.^9^,^10^ The current TB drug development portfolio capitalises on validated, druggable intracellular pathways involved in the synthesis of mycobacterial cell envelope components and include TBA-7371,^11^ BTZ043,^12^ PBTZ169 13 and OPC-167832 14,15 that all kill *Mtb* by inhibition of arabinan biosynthesis.

**Fig. 1:**
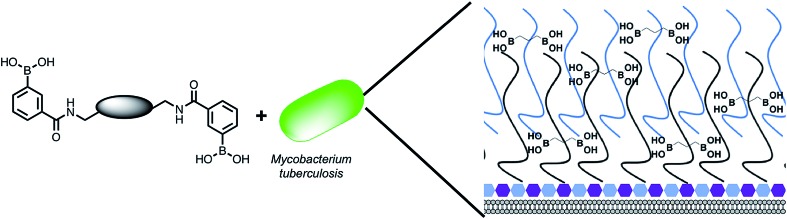
Overview of the approach used in this study. Illustration highlighting the design of multimeric boronic acids (**4–8**, Fig. 2) to specifically target the extracellular *Mtb* cell-envelope glycans. The complex *Mtb* cell envelope is simplified by black and blue lines, hexagons represent the peptidoglycan layer. This approach has several advantages over current strategies that include: no requirement for the molecule to cross the impenetrable *Mtb* cell wall barrier and the potential avoidance of drug efflux challenges.

The *Mtb* cell wall glycans are essential for its survival and pathogenesis^4^,^16^–^19^ and any disruption of the macromolecular complex can be lethal to the survival of the pathogen.^12^,^19^,^20^ Therefore, there is the tantalizing potential that these pathogen specific extracellular cell envelope glycans may, themselves, be viable therapeutic targets and afford a strategy for overcoming the intrinsic *Mtb* cell envelope barrier. To evaluate this idea further (Fig. 1), we selected to develop synthetic glycan receptors with selectivity for *Mtb* glycans as they are highly tunable and more stable under physiological conditions than biological counterparts. We selected to exploit the boronic acid pharmacophore that is an established glycan-binding functional group and forms bonds with cis 1,2 and 1,3 diols that are present in carbohydrates and there are reports of increased, synergistic binding affinity when multiple boronic acids are placed on a single scaffold.^21^–^24^ In addition, a number of boron containing agents are compatible for use in humans with a number of FDA approved boron-drugs in clinical use in other disease areas.^25^–^27^

We report here a new class of multimeric boronic acids which specifically kill *Mtb* through specific binding to *Mtb* cell envelope glycans. The most active compounds selectively kill mycobacteria over other strains of bacteria and exhibited low cytotoxicity to a panel of human cells. Whole-cell proteomics reveals a broad physiological stress response that does not result in the generation of resistance. The separation distance between the boronic acids was shown to be crucial for both activity and selectivity and evidence for preferential binding to *Mtb* associated glycans is presented. These findings suggest that new classes of anti-tubercular therapies based on targeting the unique extracellular components are possible.

## Results and discussion

### Design and synthesis of multimeric boronic acids

**Fig. 2:**
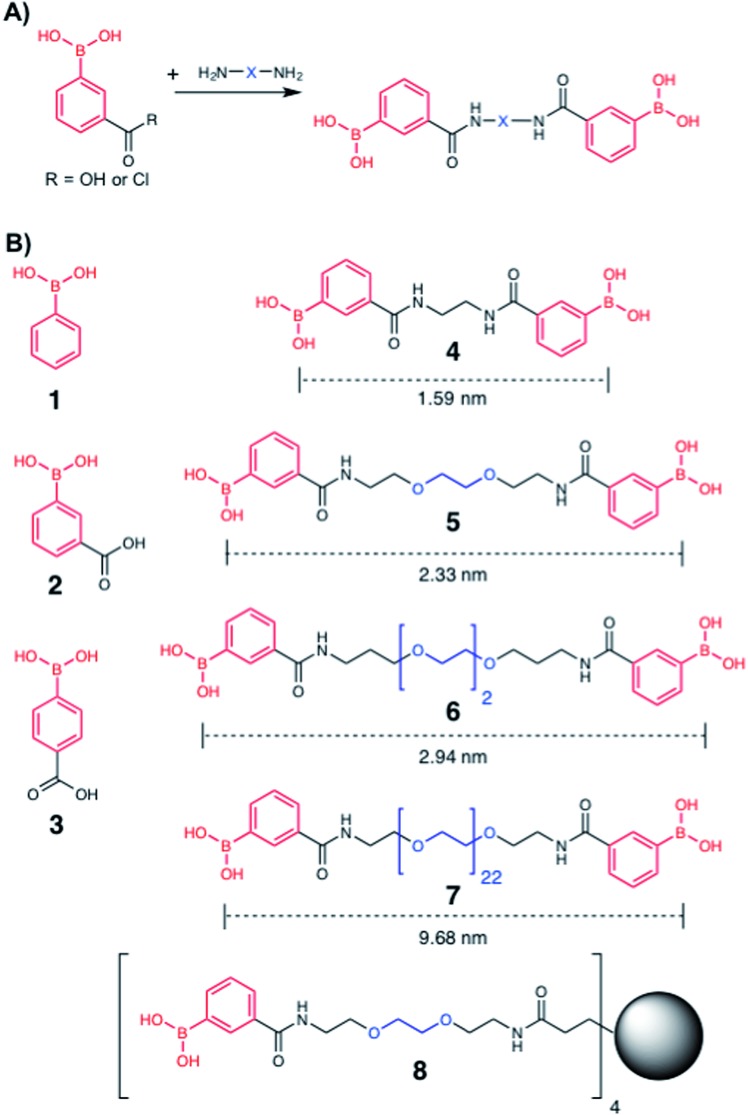
Boronic acid panel. (A) Overview of synthetic approach; (B) **1–3** were commercially available. **4–8** were synthesised as detailed in the ESI.† Distances indicate the boron–boron distances assuming a fully extended chain: angles of 109° and bond distances of 0.15 nm. Black circle is G1 PAMAM dendrimer.

To explore the potential of multivalent boronic acid analogues to selectively target *Mtb* glycans, we designed and synthesised a panel of compounds (**1–8**) to evaluate the effect of the number and separation of boronic acids units to selectively target *Mtb* glycans and kill *Mtb*. A range of compounds bearing the glycan-targeting unit, 3-carboxy-phenyl boronic acid (3-CPBA), were synthesised (**4–8**) using acid chloride or carbodiimide coupling with the appropriate di- or tetra-PEG (poly(ethylene glycol)) amine to provide flexibility of the spacer group between the relative position of the boronic acid functional groups (ESI Schemes 1–3†), giving the focused panel shown in Fig. 2 (details provided in ESI, Schemes S1–S3 and Fig. S9–24†). Systematic variation of the distance between the boronic acids (∼1.5–10 nm) was achieved using variable lengths of PEG (poly(ethylene glycol)) diamines (**5–7**). Compound **4** had an ethyl linker as a further control, but longer alkyl chains were not soluble. For **8**, a first generation PAMAM (polyamidoamine) dendrimer core was synthesised and used to generate a tetrameric boronic acid (ESI, Scheme S3†). Systematic variation of the distance between the boronic acids (1.6–10 nm) was achieved using variable lengths of PEG (**5–7**). To ensure the boronic acids were accessible for glycan binding the Alizarin red (ARS) assay was employed to map the selectivity of the dimeric boronic acids against a panel of carbohydrates and were found to retain the same affinity trends as the monomeric boronic acids (Fig. S1†).

### Determination of antibacterial potency

**Fig. 3:**
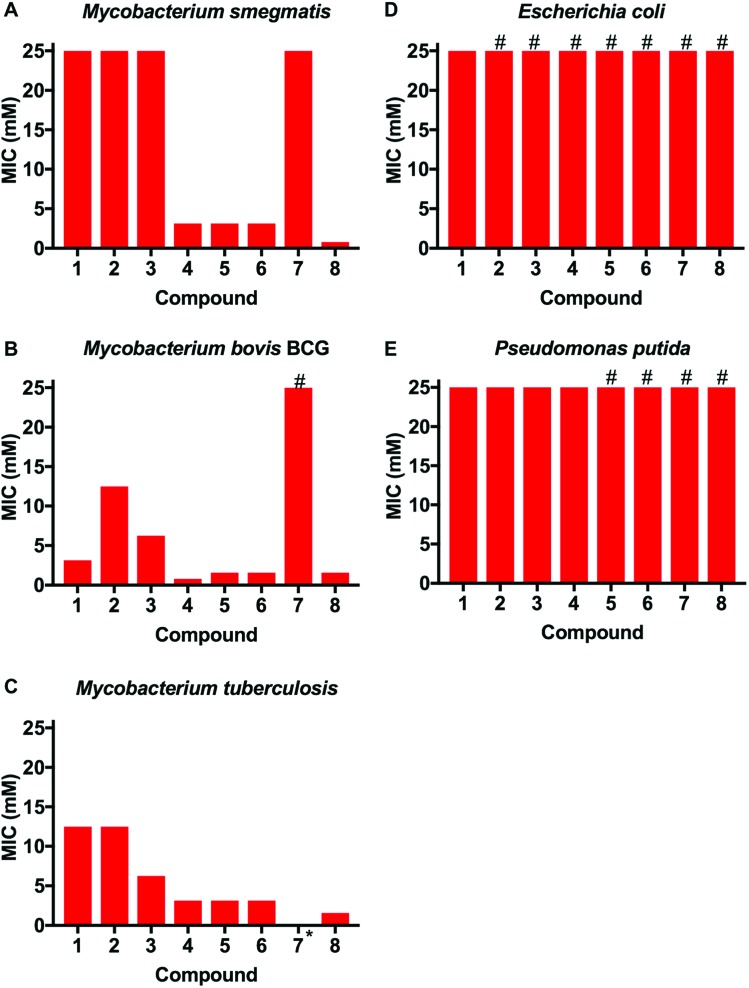
Antimicrobial activities of boronic acid derivatives **1–8** against (A) *M. smegmatis*, (B) *M. bovis* BCG, (C) *M. tuberculosis*, (D) *E. coli* (E) *P. putida*. *, not tested; # represents MIC value greater than the maximum value tested (>25 mM).

This library of glycan-targeting compounds was evaluated for antibacterial activity using the resazurin-reduction assay^28^ to determine the minimum inhibitory concentrations (MIC) (Fig. 3 and Table 1). The Gram-negative organisms *Escherichia coli* and *Pseudomonas putida* do not display the complex cell wall glycans of mycobacteria, and were tested alongside *Mycobacterium smegmatis*, *Mycobacterium bovis* BCG and also against *Mtb*. The monomeric boronic acids (**1–3**) displayed low antibacterial potency with no selective preference for Gram-negative or mycobacterial strains. Remarkably, exposure of the same strains to the dimeric boronic acids **4–6**, which vary in distance between the boronic acids from 12–23 atoms (1.6–3 nm), resulted in a dramatic decrease in the MICs (780–3100 μM against mycobacteria, Fig. 3 and Table 1) and a corresponding increase in selectivity for mycobacteria compared to Gram-negative organisms. Notably, the dimeric compounds **4–6** were more effective against *Mtb* and *M. bovis* BCG than the widely used non-pathogenic model organism *M. smegmatis*, which is faster growing. Compound **7**, with a longer linker (∼10 nm) was ineffective, with higher MICs and loss of specificity for mycobacteria. Multivalent presentation of boronic acids has been reported to lead to increased binding affinities,^22^ and the nature and length of the linker has been demonstrated in other studies to have a large influence on boronic acid binding selectivity and affinity and is likely to be important here.^23^ The increased activity of the shorter linkers would support a chelation mechanism to the cell glycans (see below for experimental evidence of binding). The longer linkers may prevent the boronic acids aligning to the bacteria surface to enable chelation, as well as a having a greater entropic penalty upon binding compared to shorter linkers, as is seen in other multivalent systems.^29^,^30^ Compound **8**, based on a 1^st^ generation poly(amidoamine) dendrimer, also showed mycobacterial selectivity with similar MIC values to the dimers **4–6** (MIC 780 μM, against *Mtb*). These results indicate that an optimal spacer length of 1.6–3 nm (compounds **4–6**) between the two boronic acid moieties is optimal whereas longer lengths >9 nm (**7**) reduce potency.

**Table 1:** Bacterial susceptibility as measured by resazurin reduction microtitre plate assay

	MIC Mycobacteria (mM)			MIC Gram-negative (mM)	
	*Mycobacterium smegmatis*	*Mycobacterium bovis BCG*	*Mycobacterium tuberculosis*	*Escherichia coli*	*Pseudomonas putida*
**1**	12.5–25	3.13	6.25–12.5	25	12.5–25
**2**	12.5–25	12.5	6.25–12.5	>25	12.5–25
**3**	25	6.25	6.25	>25	25
**4**	3.13	0.78	1.56–3.13	>25	>25
**5**	3.13	0.78–1.56	1.56–3.13	>25	>25
**6**	3.13	0.78–1.56	1.56–3.13	>25	>25
**7**	25	>25	—	>25	>25
**8**	0.78	1.56	0.78–1.56	>25	>25

Minimal bactericidal concentrations (MBCs) were also determined against *Mtb* for **4–6**. The MBC data demonstrates that these boronic acid dimers are bactericidal against actively growing *Mtb* at concentrations of 6.25 mM (Table S1†).

### *In vitro* cytotoxicity

The panel of boronic acids showed no significant cytotoxicity against human lung A549 cells, human T-lymphocyte Jurkat cells and human epithelial Caco-2 cells with MIC_99_ values above 25 mM, and no lysis or agglutination of red blood cells was observed at concentrations as high as 25 mM supporting a unique mode of action against mycobacteria and a window of activity (Table S2†). To determine if the dimeric boronic acids **4–7** were impacting surface glycans, a lectin-based hemagglutination assay was used. This determined that these dimeric boronic acids do not inhibit lectin (ConA) mediated agglutination, (Table S2 and Fig. S2†) providing further evidence that these dimeric boronic acids do not target, or bind strongly to, key glycan modifications found on mammalian cells. The active dimers (**4–6**) and tetramer **8** therefore show higher selectivity for mycobacteria compared to the mammalian cells tested that have distinct cell-surface glycans.

### Lack of cross-resistance between dimeric boronic acid analogues and rifampicin and meropenem

To evaluate if the most active compounds (**4–6**) interacted with the front-line anti-tubercular agent rifampicin a checkerboard assay was used. No synergistic or antagonistic effects were noted with the sum of the fractional inhibitory index (ΣFIC) calculated as 2. Mono-boronic acids have been reported as having β-lactamase inhibitory activity^31^,^32^ and we therefore evaluated boronic acid dimers **4–6** for compound interactions with the β-lactam meropenem on *Mtb*. The ΣFIC for each combination was calculated^33^ and found to be 1 for compound **4** and 0.6 for compounds **5–6** indicating no synergetic and importantly no antagonistic action on the growth inhibition, compared to the synergistic activity of meropenem in combination with the β-lactamase inhibitor sulbactam with a ΣFIC 0.3 (Table S3†). These observations provide evidence that the multivalent boronic acids have a unique mechanism of action compared to the monomeric boronic acid β-lactamase transition state inhibitors and are not inhibitors of mycobacterial β-lactamase targets. Guided by the above results, we attempted to obtain resistant mutants of *M. bovis* BCG when plating on 5× MIC compound **5** but were unable to obtain mutants over a period of 3 months. This can be indicative that these dimeric compounds have a multifaceted mode of action. A low level of resistance has been found for antibiotics that target cell wall precursors including vancomycin, which took over 30 years for resistance to occur,^34^ and the newly discovered texiobactin.^35^

### Identification of *Mtb* glycans as targets of dimeric boronic acids

**Fig. 4:**
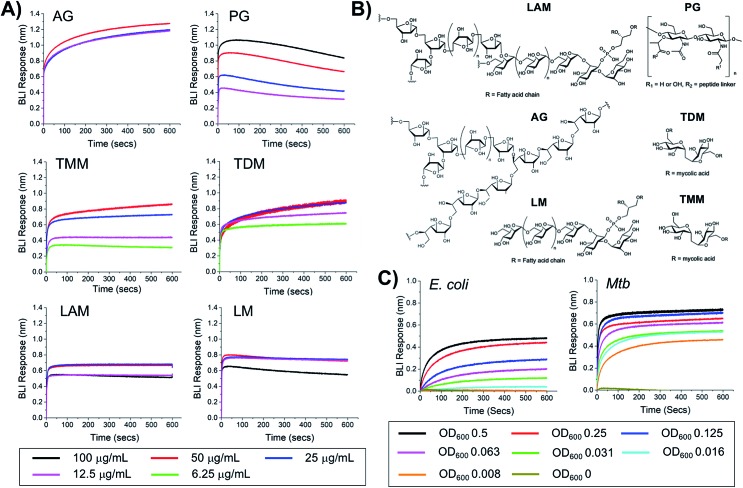
Biolayer interferometry analysis of 3-carboxy boronic acid functional sensor (A) against isolated *Mtb* cell envelope components, (B) structure of *Mtb* cell-envelope components, (C) against whole *E. coli* and (gamma-irradiated) *Mtb* cells.

To probe the selectivity and affinity of the boronic acids towards *Mtb* glycans biolayer interferometry (BLI) was employed (Fig. 4). A biotinylated boronic acid was synthesised (Scheme S4†) and immobilised onto streptavidin functionalised sensors to generate a multivalent boronic acid surface. In control experiments against dextran and galactan that do not contain *cis*-diols, and are absent from the *Mtb* cell envelope, no binding was observed (Fig. S5†). A panel of isolated *Mtb* cell envelope components (Fig. 4) (additional information in ESI Fig. S3–S5†) were subsequently evaluated by BLI, revealing that the boronic acids interacted strongly with *Mtb* components that contain glycans with *cis*-diols (Fig. 4, S3 and S4†): PG, AG, TMM, TDM, LAM and LM. *K*_d_ values were obtained using a steady-state model, giving values of 41 μg mL^–1^ (PG), 4 μM (TDM), 12 μM (TMM) and 41.5 μg mL^–1^ (LAM) (Table 2). Despite strong binding observed to AG we were unable to calculate the *K*_d_ value as saturation was not reached. In comparison, weak binding affinity towards PIM6 (which has six mannoses decorating the PIM unit) was observed, and very weak binding for PIM 1 + 2 (which have correspondingly lower degrees of glycosylation) and notably, no detectable binding for isolated mycolic acid methyl esters (MAMEs) and sulpholipid I which contains a sulphated trehalose moiety. This is consistent with a multivalent interaction between cell wall components that contain *cis*-diols and the boronic acid and clearly demonstrates that boronic acids have the necessary capacity to engage with these essential *Mtb* cell envelope constituents. To further determine the origin of the specificity of the multimeric boronic acids for mycobacterial glycans, we probed the interaction of the boronic acid functionalised sensor with mammalian specific glycans using GM3 (monosialodihexosylganglioside), which contains a galactose residue in the pyranose conformation linked to a terminal sialic acid moiety, and no detectable binding was observed (Fig. S6†). Whole *E. coli* and (gamma-irradiated) *Mtb* cells were evaluated by BLI for binding to the boronic acid functionalised sensors. *Mtb* gave significantly faster rates and extent of binding compared to *E. coli* (Fig. 4C). To evaluate this further, we investigated the interaction of lipopolysaccharide (LPS) from *E. coli*, which is a major cell wall component located on the outer membrane of Gram-negative organisms, by BLI and weak binding was observed (Fig. S6†), consistent with the observed interactions of whole *E. coli* cells. Taken together, this BLI data supports a hypothesis that multimeric boronic acids selectively target mycobacterial cell wall glycans that are not found in other bacterial species or mammalian cells, leading to the observed bactericidal activity. The lack of affinity for sialic acid terminated glycans, which features as a terminal modification on cell-surface glycans in mammalian cells may contribute to the selectivity observed here.

**Table 2:** Binding affinities from BLI

	*K*_d_
PG	41 μg mL^–1^
TDM	4 μM
TMM	12 μM
LAM	41.5 μg mL^–1^

### Global protein expression response of *M. bovis* BCG to dimeric boronic acids

**Fig. 5:**
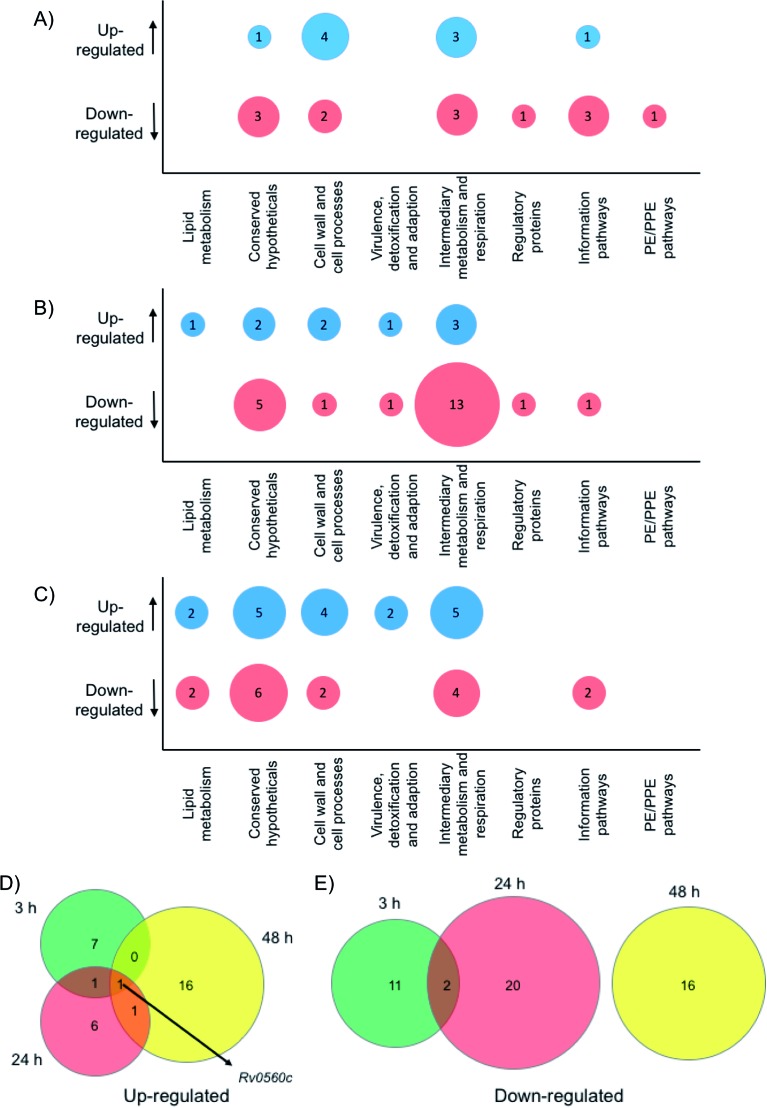
Whole cell proteomic analysis in response to **6**. Bubble plots show *Mtb* proteins that are up- or down-regulated at (A) 3 hours, (B) 24 hours and (C) 48 hour. Venn diagrams indicate the number of proteins that are (D) up-regulated or (E) down-regulated after 3 hours (green), 24 hours (red) or 48 hours (yellow) exposure to **6**.

To gain physiological insight into the mode of action of these multimeric boronic acids whole cell proteomics was employed. Monoboronic acids are known to target intracellular penicillin binding proteins, β-lactamases, proteases and tRNA synthetase proteins^31^,^32^,^36^–^38^ and we wanted to compare and characterise the impact of multimeric boronic acid analogues on the mycobacterial proteome. *M. bovis* BCG was exposed to 2× MIC of boronic acid dimer **6** and analysis of the whole cell protein expression profile at 3 hours, 24 hours and 48 hours was performed. A list for all the identified proteins, annotations and fold changes compared to controls at each time point are in ESI List S1 (Fig. 5 and S8†).

Crucially, we detected no up- or down-regulation of penicillin binding proteins, β-lactamases, proteases and tRNA synthetase proteins (ESI List S1†) that have been previously implicated as targets for mono-boronic acids^31^,^32^,^36^–^38^ enabling us to rule out these intracellular targets, in-line with the meropenem checkerboard assays (Fig. S7 and Table S3†). Interestingly, proteins from two functional groups: category 3 (cell wall and cell processes) and category 7 (intermediary metabolism and respiration) were particularly affected, indicating that *Mtb* has a general stress response upon exposure of the dimeric boronic acids. In general, we found little overlap in the proteins that were either up- or down-regulated at the different time points (Fig. 5). However it was particularly notable that one identical protein that is involved in intermediary metabolism and respiration: Rv0560c, a putative benzoquinone methyltransferase that has been associated with an increase in oxygen consumption by *Mtb* and is an indicator of a stress response^39^,^40^ was upregulated at all time points. It is likely that chelation of the multimeric boronic acids to the essential mycobacterial glycans disrupts essential physiological processes that are lethal to *Mtb*. Taken together the proteomics data confirms that the multivalent presentation of boronic acids affords a new route to kill *Mtb*.

## Conclusions

In conclusion, here we have introduced a new approach to selectively kill *Mtb* by chelation of its unique cell wall glycans using multivalent boronic acids. This is conceptually distinct from existing drugs, which target defined intracellular pathways and hence must also permeate the *Mtb* cell envelope that confers intrinsic resistance to many antibiotics. The multivalent boronic acids were shown to selectively kill mycobacteria over other bacterial species. The distance between boronic acids was crucial with longer linkers reducing activity and selectivity. Two boronic acid units were optimal, with a tetramer (**8**) showing almost identical activity to the dimers. Biolayer interferometry revealed strong and selective interactions with isolated *Mtb* glycans and whole intact *Mtb* cells and whole cell proteomics identified a broad stress response rather than a single target, which may contribute to the lack of resistance observed. The multimeric display of boronic acids was crucial to their mechanism of action and distinct function compared to analogous monovalent boronic acids. This concept of inhibiting the extracellular glycans on *Mtb* presents a unique opportunity to develop pathogen specific agents and represents an important step in the identification of new TB drug targets.

## Conflicts of interest

The authors (CSG, MIG and EF) are named inventors on a patent application relating to this work.

## Supplementary Material

Supplementary information

Supplementary information
